# The types of school attended by the ALSPAC children from1997 to 2011 (ages 5 to 19 years): A Focus on Christian Faith Schools.

**DOI:** 10.12688/wellcomeopenres.23025.1

**Published:** 2025-02-27

**Authors:** Hamid Reza Tohidinik, Mark Mumme, Jean Golding

**Affiliations:** 1 Centre for Academic Child Health, Population Health Sciences, Bristol Medical School, University of Bristol, Bristol, England, BS8 2PS, UK; 2 Avon Longitudinal Study of Parents and Children, Population Health Sciences, Bristol Medical School, University of Bristol, Bristol, England, BS8 2BN, UK

**Keywords:** School characteristics, ALSPAC, Christian Faith schools, Children, Religious schools, Anglican schools, Roman Catholic schools, UK.

## Abstract

The Avon Longitudinal Study of Parents and Children (ALSPAC) is a longitudinal study following ~14,000 children from pregnancy through until adulthood. They were all born to women resident during pregnancy in a geographic area which comprised the city of Bristol, surrounding suburbs, rural areas, villages and towns. During their childhood almost all attended either state or private schools. The present Data Note describes the basic details of the schools attended by the cohort of children born in 1991-2, obtained by linking the names of the cohort children to the schools they attended during each school year and then anonymising the data. Details include the size of school in terms of the number of children enrolled, school sex composition, whether it is a Christian faith school (including the type of faith), whether it is fee-paying, and whether it is a boarding school. This document includes details as to how scientists can obtain the data for analysis in regard to other aspects of the children involved.

## Introduction

The environment in which children grow and develop has a profound impact on their overall well-being/health and future prospects. Among the various components of this environment, schools hold a particularly influential position by playing a pivotal role in shaping their physical, mental, and social health as well as their educational development ( Edgerton & McKechnie, 2022; Miyazaki & Stack, 2015; Pallan *et al.*, 2014; Patalay *et al.*, 2020).

Schools, often considered as a second home for children, serve as more than just centres for education; they provide a context in which children develop key life skills, establish social relationships ( Pacheco *et al.*, 2022), and learn healthy behaviours ( Morton *et al.*, 2016).

To comprehend the complex interplay between school characteristics, child and adolescent health, and educational achievement, it is useful to encompass a diverse range of school attributes in population health studies. This is particularly pertinent in longitudinal studies, as it enables researchers to comprehensively assess their influence on shaping the trajectory of children's lives while minimizing potential bias. Nevertheless, the backgrounds of the schools are rarely considered in studies of health and development (apart from academic achievements ( Edgerton & McKechnie, 2022)).

One of the least studied school identities concerns the contribution of faith schools. In England the number of Church of England (C of E) faith schools started expanding in 2001, resulting in considerable controversy. By 2004, about one-third of primary, and one-sixth of secondary schools were faith schools ( Cush, 2005). Yeshanew *et al.* carried out a comprehensive study comparing the academic results of faith with non-faith primary schools. They showed that the pupils in Roman Catholic and C of E schools performed slightly better than those in non-faith schools, and that pupils with special educational needs were particularly advantaged by being in a faith school ( Yeshanew *et al.*, 2008).

Although fewer in number, the secondary faith schools in England are predominantly Christian, 58% of these were Roman Catholic and 35% C of E. Allen and West (2011) showed that the social composition of attendees at the faith secondary schools varied with fewer children having free school meals but more of Black African and Afro-Caribbean backgrounds than the population attending non-faith secondary schools. They noted that there were fewer such schools in the south-west of the country ( Allen & West, 2011).

The Avon Longitudinal Study of Parents and Children (ALSPAC) is a longitudinal birth cohort with a wide range of genetic, psychological, physical, and environmental data ( Boyd *et al.*, 2013; Fraser *et al.*, 2013; Golding *et al.*, 2001; Northstone *et al.*, 2019). The aim of this data note is to provide an overview of the existing data on characteristics of schools that ALSPAC children attended during their childhood and adolescence. It provides important information for future ALSPAC users who are interested in this subject, to use it for testing their own hypotheses concerning the advantages and disadvantages of attending a Christian faith school.

## Methods

### The ALSPAC sample and the study children

ALSPAC is a longitudinal birth cohort in which all pregnant women resident in Avon, UK with expected dates of delivery between 1st April 1991 and 31st December 1992 were invited to take part. 20,248 pregnancies have been identified as being eligible and the initial number of pregnancies enrolled was 14,541. Of the initial pregnancies, there was a total of 14,676 foetuses, resulting in 14,062 live births and 13,988 children who were alive at 1 year of age. When the oldest children were approximately 7 years of age, an attempt was made to bolster the initial sample with eligible cases who had failed to join the study originally. As a result, when considering variables collected from the age of seven onwards there are data available for more than the 14,541 pregnancies mentioned above: The number of new pregnancies not in the initial sample (known as Phase I enrolment) that are currently represented in the released data and reflecting enrolment status at the age of 24 is 906, resulting in an additional 913 children being enrolled (456, 262 and 195 recruited during Phases II, III and IV respectively). The total sample size for analyses using any data collected after the age of seven is therefore 15,447 pregnancies, resulting in 15,658 foetuses. Of these 14,901 children were alive at 1 year of age. The children have been followed ever since, through infancy, toddlerhood, the pre-school, infant and primary school phases and on to secondary school and college. Here we map the features of the schools attended from years 1997 to 2011.

Further details on the ALSPAC study have been published elsewhere ( Boyd *et al.*, 2013; Fraser *et al.*, 2013; Northstone *et al.*, 2019; Golding *et al.*, 2001). The study website contains details of all data available through a fully searchable data dictionary and variable search tool: http://www.bristol.ac.uk/alspac/researchers/our-data/.

### Obtaining details of the schools attended (History of linkage of ALSPAC to education records)

***Early education data linkage – before the Project to Enhance ALSPAC through Record Linkage (PEARL)***

Permission to link to the externally collected educational and medical records of the index children participating in ALSPAC was originally sought from the accompanying adult at a study-data collection ‘clinic’ run between September 1998 and September 2000, called the ‘Focus@7’ clinic. In total, 7,843 out of the 13,146 children recruited during the initial phase of ALSPAC and enrolled at the time attended this clinic. Additionally, 456 children who attended were recruited into ALSPAC after the initial phase. Therefore, in May 2003, the ALSPAC Ethics & Law Committee (ALEC) reviewed the approach and decided that, in line with the Data Protection Act, it was not necessary for ALSPAC to obtain written consent before using the data, but that data on children where parents objected at the Focus@7 clinic - or for whom permission was subsequently withdrawn - should not be requested. In total 15 objections were registered at this stage (14 at the clinic visit, and 1 subsequently).

Following the decision by ALEC, the four Local Education Authorities (LEA) that covered the former Avon area (Bristol, South Gloucestershire, Bath & North East Somerset and North Somerset) were approached and agreed that ALSPAC would be allowed to access these data. The School Entry Assessment and Key Stage 1 records were first linked to each other using the LEA PIN numbers and name, date of birth and sex. This was done by ALSPAC’s data linkage team using a deterministic linkage methodology. The resulting master list of children was then linked to ALSPAC identifiers using names and dates of birth. This early education dataset was initially made available by ALSPAC for research in early 2004.

Subsequently, the Department for Education (DfE) (or the Department for Children, Schools and Families (DCSF) as it was until 2010) introduced the National Pupil Database (NPD). The NPD was established in 2002 (but includes records going back to academic year 1995/1996) and is a longitudinal database of pupils who attend state-maintained schools and colleges. The NPD holds detailed information about children’s education at different stages, exam and test results, absence and exclusion data, as well as pupil characteristic data such as ethnicity, language, free school meals entitlement, and details of any Special Educational Needs (SEN). The DfE introduced the Unique Pupil Number (UPN) in 1999 to facilitate the collection of data across the school system; later updating this to the Pupil Matching Reference–anonymous (PMRa). This enables the linkage of participants’ identifiers to a systematically compiled pupil register.

In 2002, ALSPAC obtained these linked NPD records for the ALSPAC participants. In order to satisfy the confidentiality requirements of both ALSPAC and the DfE, the linking work was carried out by an independent charity, The Fischer Family Trust (FFT), acting as a trusted third party. ALSPAC supplied the FFT with names, dates of birth and current postcode for all members of the eligible cohort, whilst DfE supplied similar details relating to all the individual datasets in the NPD. The FFT then linked ALSPAC identifiers to the DfE’s UPN by matching the available data separately for each data set (i.e., separate matches for each of the several datasets held and maintained by the DfE). This occasionally resulted in a single ALSPAC child being linked to different pupils in different years/datasets. Such cases were subject to quality checks conducted by ALSPAC to validate the linkage. In addition, a small number of bad matches were identified by investigating cases for whom the reported national curriculum year was 2 or more years out from the expected national curriculum year.

ALSPAC obtained updates from the DfE for Key Stage 1 (KS1), Key Stage 2 (KS2, first made available by ALSPAC from 2008), Key Stage 3 (KS3, also available from 2008) and Key Stage 4 (available from 2010) at a national level (England) replacing the datasets previously linked at a local authority level. The School Entry Assessment data collected locally was not updated as these data are not held in the NPD. These updates were obtained using the existing UPN, as established by FFT, with the data of those who dissented removed.

## Ethical approval and consent

Ethical approval for the study was obtained from the ALSPAC Law and Ethics Committee (ALEC; IRB00003312; date: 01-06-2010) and the local research ethics committees (NHS Haydock REC: 10/H1010/70; date: 03-02-2011). The education data was provided by the DfE following their internal decision-making process, under Data Request Number DR120430.04 (dated 30-04-2012), which states that 'ALSPAC operates as a resource for the entire research community’ and 'The [education] data are pseudonymised and then used by researchers and research projects approved by the ALSPAC executive.’ Consent for the use of data collected via questionnaires (implied consent) and clinics (informed consent) was obtained from participants following the recommendations of the ALSPAC Ethics and Law Committee at the time. Questionnaires were completed in the participants’ own home and return of the questionnaires was taken as continued consent for their data to be included in the study ( Birmingham, 2018). Full details of the approvals are available from the study website. Study members have the right to withdraw their consent for elements of the study or from the study entirely at any time.

### PEARL consent campaign

**Figure 1.**
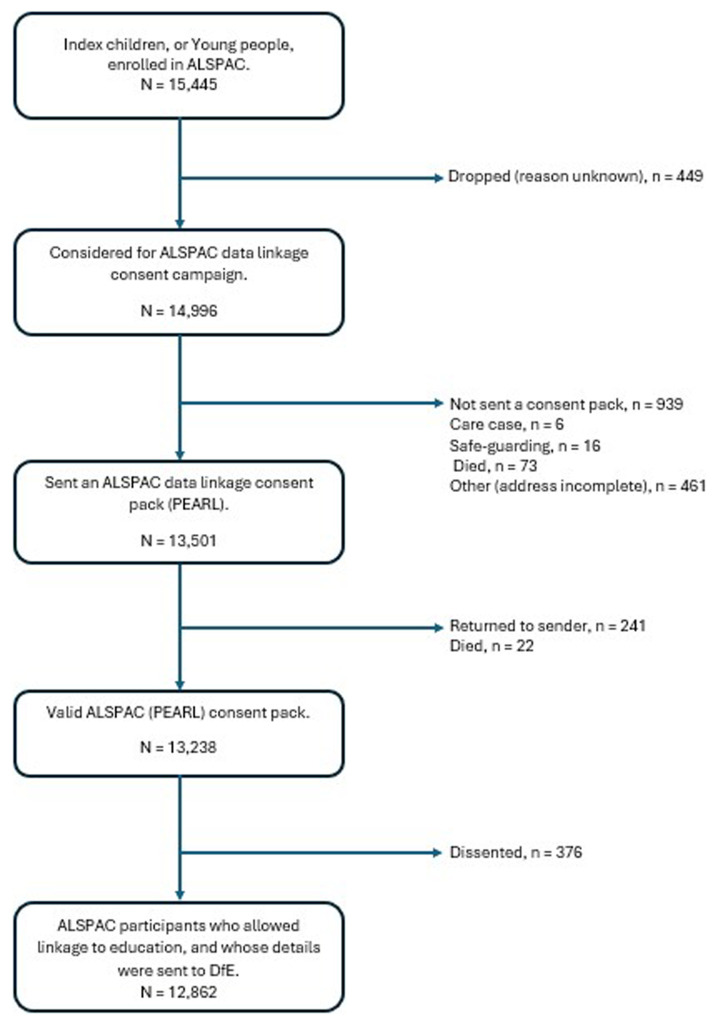
Flowchart of school data linkage for ALSPAC children.

When the ALSPAC children reached legal adulthood (age 18 years), the ALSPAC data linkage team, through the Project to Enhance ALSPAC through Record Linkage (PEARL), sent them 'fair processing' materials describing ALSPAC’s intention to link to their routine health and administrative data from national databases, including education, and gave them a clear means to consent or object via a written form. Participants were informed that ALSPAC would attempt to link to their records unless they opted out. Data were not extracted for participants who objected, or who were not sent fair processing materials. The enrolled sample of the ALSPAC cohort at this stage consisted of 15,445 individuals, and 14,996 of these were initially included in the PEARL consent campaign. The PEARL campaign sent out a total of 13,501 consent packs to the ALSPAC young people (the children now being young adults in their own right). Participants were asked to consider allowing data linkage to health, education, crime and environmental data. The education was split into compulsory schooling, further education and higher education, and the participants could allow, or opt out of, any or all of these three categories. There were 263 consent packs which were known not to have been received by the participant. A record was made of those participants who did not receive the pack (e.g. item returned to sender). At the time these data were obtained there were 12,862 participants who had allowed their education records from DfE to be accessed by ALSPAC. Note that these consent figures represent a point in time; consent is ongoing, and numbers have changed over time. The summary of the current school data linkage for ALSPAC children is presented in Figure 1.

The available data set comprises those who explicitly consented together with those who chose not to opt-out. Those who either dissented or were not provided with a consent pack have had their data removed and set to missing.

The ALSPAC extract of the NPD data comprises datasets from the academic years 1997/1998 to 2011/2012. The schools in this dataset are those recorded as the school at which the participants completed their Key Stage assessments, Key Stages 1 to 5, and are drawn from the DfE’s Key Stage attainment datasets. The number of ALSPAC participants who were identified and matched by the DfE at each Key Stage are presented in Table 1.

**Table 1.** The number of ALSPAC participants who were identified and matched by the DfE at each Key Stage.

Key Stage (KS)	Number
KS1	11,279
KS2	12,233
KS3	10,851
KS4	11,764
KS5	9,449

ALSPAC: The Avon Longitudinal Study of Parents and Children.

Variables:

The school data were converted from long format to wide format to capture the student's school characteristics at different years separately and link them to the ALSPAC core data for further analysis. The school variables utilized in this paper are listed in Table 2.

**Table 2.** School data variables linked to ALSPAC used in this study.

Variable	Variable name in school data linked to ALSPAC	Variable coding
Activity level	activity_level1997 to activity_level2011	KS1 vs KS2 vs KS3 vs KS4 vs KS5
Religious category of the schools	religious_character1997 to religious_character2011	Anglican vs Roman Catholic vs Other Christian vs Other (Non faith, Jewish, and Islam)
Phase of education	phase_of_education1997 to phase_of_education2011	Primary vs Secondary vs All-Through vs 16 Plus vs Not Applicable
Boarding status	boarders_situation1997 to boarders_situation2011	Boarding School vs College/FE Residential Accommodation vs Children's Home (Boarding School) vs No Boarders vs Not Applicable
Establishment type	establishment_type1997 to establishment_type2011	Colleges vs Local Authority Maintained School vs Special School vs Independent School vs Other
Gender composition	gender_of_pupils1997 to gender_of_pupils2011	Mixed vs Girls only vs Boys only
Duration of attending Christian faith schools	religious_school_years (derived variable)	Continuous (Years)
Attended Christian faith school ever	religious_school_binary (derived variable)	Binary (Non-faith vs at least one Christian faith school in life.
Level of attendance at a Christian faith school	Faith_school_level (derived variable)	Non-faith vs Primary included vs After primary only

ALSPAC: The Avon Longitudinal Study of Parents and Children.

The ALSPAC participants, as pupils, may have attended several schools throughout their education. It is also important to note that the dataset records the characteristics of the school as it was during the academic year that the DfE extract related to, and that the school may have changed over the years in some way or even closed or merged, either during the period recorded or since. The school characteristics were consolidated into groupings as shown in Table 3. All the schools were given a random study ID (study_urn1997, study_urn1998, ..., study_urn2011) which is unique for this project; so that the schools are individually labelled but cannot be re-identified.

**Table 3.** Consolidated Groupings of School Characteristics by Religious category, Establishment type, and Phase of Education.

Religious category	KS categories
Anglican	Anglican
	Anglican/Christian
	Anglican/Church of England
	Church of England
	Church of England/Methodist
	Church of England/United Reformed Church
Roman Catholic	Roman Catholic
	Roman Catholic/Church of England
	Roman Catholic/Anglican
	Catholic
	Church of England/Roman Catholic
Other Christian	Methodist
	Christian
	Anglican/Evangelical
	Church of England/Evangelical
	Protestant/Evangelical
	Seventh Day Adventist
	Plymouth Brethren Christian Church
	Quaker
Other	Jewish
	Islam
	Inter- / non- denominational
No religious character	Does not apply
	None
	[blank]
Type of Establishment	KS labelling
Colleges	Colleges
Local authority maintained schools	Local authority maintained schools
Special schools	Special schools
Independent schools	Independent schools
Other	Academies
	Other types
	Universities
	Welsh schools
Phase of Education	KS labelling
Primary	Primary
	Middle deemed primary
Secondary	Secondary
	Middle deemed secondary
All-through	All-through
16 plus	16 plus
Not applicable	Not applicable

KS: Key Stage; The categories of boarders and sex composition of pupils were not altered from the KS labelling.

## Statistical analysis

We examined the distribution of children in the schools from 1997 to 2011 according to the type of establishment, whether boarders were taken, religious categories, and sex of children in the school. In cases where the number of students was small within a specific category, we opted to merge those categories with adjacent ones for analysis. All statistical analyses were performed using Stata 17.0 (StataCorp LLC, College Station, TX, USA), licensed to the University of Bristol.

## Results

Of the 12862 children, 6488 (50.5%) were male. They predominantly attended community schools under local authority administration until the age of ~17 (in the academic year 2009–10), with the lowest proportion of records for 2009-10 (46.8%) and the highest in 1997–8 (99.1%) at ages 5–7. Subsequently, most transitioned to attending college. The majority of the students were non-boarding day pupils, accounting for at least 89.8% in 2011–12, and studied in mixed-sex schools (minimum of 96.5% in 2008–9 and 2009–10) ( Table 4). Thirty nine students attended more than one school during an academic year.

**Table 4.** Characteristics of ALSPAC children and their schools (1997–2011).

		Sex, N	Establishment type, N					Boarding status, N		Sex composition, N		
Academic year	Approximate age (years)	Female	College	Local authority	Special school	Independent school	Other	Boarding school *	No boarders	Mixed	Girls only	Boys only
1997–98	5–6	1368	-	2,683	13	10	<5	<5	2,704	2,704	<5	<5
1998–99	6–7	3622	-	7,268	19	98	<5	38	7,346	7,373	12	<5
1999–2000	7–8	1133	-	2,217	5	36	<5	15	2,242	2,253	5	<5
2000–01	8–9	<5	-	<5	<5	<5	<5	<5	<5	<5	<5	<5
2001–02	9–10	1394	-	2,607	24	133	<5	40	2,723	2,749	15	<5
2002–03	10–11	3594	-	7,032	43	286	<5	81	7,275	7,337	23	<5
2003–04	11–12	1134	-	2150	10	105	<5	26	2,238	2,250	15	<5
2004–05	12–13	1289	-	2,409	32	131	37	46	2,562	2,576	21	12
2005–06	13–14	3390	-	6,540	87	325	64	108	6,906	6,911	53	52
2006–07	14–15	1392	9	2,388	43	296	37	93	2,671	2,691	65	17
2007–08	15–16	4263	346	7,033	91	746	306	260	8,079	8,270	185	67
2008–09	16–17	3698	1,446	4,565	25	518	390	285	5,813	6,701	177	66
2009–10	17–18	3021	2,059	2,622	6	479	429	298	3,851	5,402	132	61
2010–11	18–19	1209	1,067	820	<5	185	192	124	1,306	2,199	51	16
2011–12	19–20	144	191	52	<5	12	41	13	114	288	6	<5

ALSPAC: The Avon Longitudinal Study of Parents and Children. *Merged with Children’s Home (boarding) and College/ FE residential. “<5” cells may include zero.

**Figure 2.**
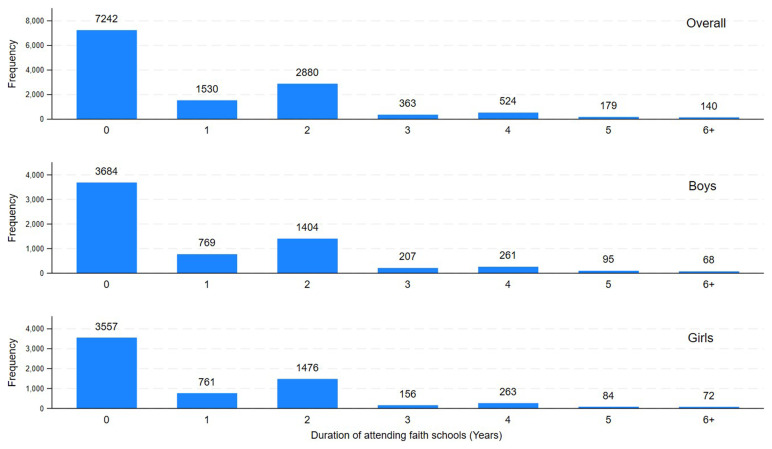
Years attended Christian faith schools by ALSPAC children overall, girls, and boys.

A majority of participants attended non-faith schools during the whole period of follow-up (minimum of 66.9% in 2002–3). Anglican schools were the predominant Christian faith schools until 2006–7 (ranged between 4.1% and 27.0%), but after that the number of students were slightly higher in Roman Catholic schools (maximum of 9.0% in 2009–10) ( Table 5). Figure 2 represents the number of years pupils attended Christian faith schools during their life with similar distributions observed among girls and boys. In total, 5616 (43.7%) attended a Christian faith school at least once in their life. Among them, 4562 (81.6%) attended a Christian faith school during primary school, while the remaining 18.4% attended a Christian faith school only after primary school. Mean duration of attending the Christian faith schools was 0.95 years (SD:1.33, Range: 0–7 years) in all children. It was 2.17 years (SD:1.17) for those who ever attended a Christian faith school. The majority of boarding schools tended to be faith schools until 2007–8, but in later years they were mostly non-faith ( Table 6). Schools’ religious characters by sex composition and establishment type are presented in Table 7 and Table 8.

**Table 5.** Religious character of schools attended by ALSPAC children, by different levels of education (1997–2011).

Year	Sex	Activity level †	School religious character, N			
			Non faith *	Anglican	Roman Catholic	Other Christian
1997–8	Boys	KS1	990	278	70	<5
	Girls	KS1	981	310	77	<5
1998–9	Boys	KS1	2732	813	212	6
	Girls	KS1	2560	833	220	9
1999–2000	Boys	KS1	829	233	59	<5
	Girls	KS1	812	243	77	<5
2000–1	Boys	KS1	<5	<5	<5	<5
	Girls	KS2	<5	<5	<5	<5
2001–2	Boys	KS2	925	365	72	8
	Girls	KS2	936	378	73	7
2002–3	Boys	KS2	2529	1017	201	20
	Girls	KS2	2395	974	209	16
2003–4	Boys	KS2	778	290	52	8
		KS3	<5	<5	<5	<5
	Girls	KS2	759	290	80	3
		KS3	<5	<5	<5	<5
2004–5	Boys	KS2	<5	<5	<5	<5
		KS3	1140	73	73	32
	Girls	KS3	1152	66	63	8
2005–6	Boys	KS3	3270	150	132	70
		KS4	<5	<5	<5	<5
	Girls	KS3	3090	139	140	20
		KS4	<5	<5	<5	<5
2006–7	Boys	KS4	1197	76	67	41
	Girls	KS4	1247	67	55	23
2007–8	Boys	KS4	3401	157	139	87
		KS5	362	47	56	10
	Girls	KS4	3336	149	144	43
		KS5	450	58	78	5
2008–9	Boys	KS4	1019	58	40	28
		KS5	1668	168	208	56
	Girls	KS4	1060	50	41	7
		KS5	2049	174	292	25
2009–10	Boys	KS4	10	<5	<5	<5
		KS5	2107	181	225	51
	Girls	KS4	<5	<5	<5	<5
		KS5	2536	172	279	30
2010–11	Boys	KS5	878	75	78	26
	Girls	KS5	1057	52	93	7
2011–12	Boys	KS5	132	8	9	<5
	Girls	KS5	136	<5	5	<5

ALSPAC: The Avon Longitudinal Study of Parents and Children; KS: Key Stage; †Activity level indicates the Key Stage assessments completed by the participants; *Includes a small number of pupils who attended Muslim faith schools; “<5” cells may include zero.

**Table 6.** Religious character of schools attended by ALSPAC children, by boarding status (1997–2011).

Year	Boarding status	School religious character, N			
		Non faith *	Anglican	Roman Catholic	Other Christian
1997–8	Boarding school	<5	<5	<5	<5
	No boarders	1969	588	147	<5
1998–9	Boarding school	14	20	<5	<5
	No boarders	5277	1626	432	11
1999–2000	Boarding school	5	8	<5	<5
	No boarders	1636	468	136	<5
2000–1	Boarding school	<5	<5	<5	<5
	No boarders	<5	<5	<5	<5
2001–2	Boarding school	14	18	<5	8
	No boarders	1846	725	145	7
2002–3	Boarding school	28	29	<5	24
	No boarders	4892	1961	410	12
2003–4	Boarding school	7	12	<5	7
	No boarders	1532	569	133	<5
2004–5	Boarding school	13	22	<5	42
	No boarders	2280	117	132	33
2005–6	Boarding school	34	49	8	17
	No boarders	6329	240	264	73
2006–7	Boarding school	35	28	5	25
	No boarders	2400	115	117	39
2007–8	Boarding school	116	64	11	69
	No boarders	7250	347	406	76
2008–9	Boarding school	161	68	<5	52
	No boarders	4791	382	576	64
2009–10	Boarding school	180	68	8	42
	No boarders	3034	285	493	39
2010–11	Boarding school	79	30	<5	14
	No boarders	1021	97	169	19
2011–2	Boarding school	7	5	<5	<5
	No boarders	92	6	14	<5

ALSPAC: The Avon Longitudinal Study of Parents and Children. * Includes a small number of pupils who attended Muslim faith schools; “<5” cells may include zero.

**Table 7.** Religious character of schools attended by ALSPAC children, by school sex composition (1997–2011).

Year	School sex composition	School religious character, N			
		Non faith *	Anglican	Roman Catholic	Other Christian
1997–8	Mixed	1969	588	147	<5
	Unisex	<5	<5	<5	<5
1998–9	Mixed	5280	1646	432	15
	Unisex	12	<5	<5	<5
1999–2000	Mixed	1637	476	136	<5
	Unisex	5	<5	<5	<5
2000–1	Mixed	<5	<5	<5	<5
	Unisex	<5	<5	<5	<5
2001–2	Mixed	1846	743	145	15
	Unisex	15	<5	<5	<5
2002–3	Mixed	4902	1990	409	36
	Unisex	22	<5	<5	<5
2003–4	Mixed	1526	580	133	11
	Unisex	14	<5	<5	<5
2004–5	Mixed	2265	137	134	40
	Unisex	29	<5	<5	<5
2005–6	Mixed	6268	285	270	88
	Unisex	97	<5	<5	<5
2006–7	Mixed	2368	140	120	63
	Unisex	76	<5	<5	<5
2007–8	Mixed	7314	404	414	138
	Unisex	235	7	<5	7
2008–9	Mixed	5571	446	576	108
	Unisex	226	<5	5	8
2009–10	Mixed	4478	348	502	74
	Unisex	179	5	<5	7
2010–11	Mixed	1871	127	169	32
	Unisex	64	<5	<5	<5
2011–2	Mixed	260	11	14	<5
	Unisex	8	<5	<5	<5

ALSPAC: The Avon Longitudinal Study of Parents and Children. * Includes a small number of pupils who attended Muslim faith schools; “<5” cells may include zero.

**Table 8.** Religious character of schools attended by ALSPAC children, by establishment type (1997–2011).

Academic Year	Establishment type	School religious character, N			
		Non faith *	Anglican	Roman Catholic	Other Christian
1997–8	Local authority	1948	588	147	<5
	Special school	13	<5	<5	<5
	Independent school	10	<5	<5	<5
1998–9	Local authority	5211	1624	432	<5
	Special school	19	<5	<5	<5
	Independent school	62	22	<5	14
1999–2000	Local authority	1613	468	136	<5
	Special school	5	<5	<5	<5
	Independent school	24	8	<5	<5
2000–1	Local authority	<5	<5	<5	<5
	Special school	<5	<5	<5	<5
	Independent school	<5	<5	<5	<5
	Other	<5	<5	<5	<5
2001–2	Local authority	1739	723	145	<5
	Special school	24	<5	<5	<5
	Independent school	98	20	<5	15
2002–3	Local authority	4664	1959	407	<5
	Special school	43	<5	<5	<5
	Independent school	217	32	<5	34
2003–4	Local authority	1451	567	132	<5
	Special school	10	<5	<5	<5
	Independent school	79	14	<5	11
2004–5	Local authority	2159	117	132	<5
	Special school	32	<5	<5	<5
	Independent school	66	22	<5	39
	Other	37	<5	<5	<5
2005–6	Local authority	6025	245	264	6
	Special school	87	<5	<5	<5
	Independent school	189	44	8	84
	Other	64	<5	<5	<5
2006–7	College	8	<5	<5	<5
	Local authority	2157	114	116	<5
	Special school	43	<5	<5	<5
	Independent school	199	29	5	63
	Other	37	<5	<5	<5
2007–8	College	219	<5	127	<5
	Local authority	6407	345	275	6
	Special school	90	<5	<5	<5
	Independent school	528	65	14	139
	Other	305	<5	<5	<5
2008–9	College	971	<5	475	<5
	Local authority	4092	366	102	5
	Special school	25	<5	<5	<5
	Independent school	340	63	<5	111
	Other	369	21	<5	<5
2009–10	College	1588	<5	471	<5
	Local authority	2340	254	23	5
	Special school	6	<5	<5	<5
	Independent school	329	65	10	75
	Other	394	34	<5	<5
2010–11	College	905	<5	162	<5
	Local authority	725	87	8	<5
	Special school	<5	<5	<5	<5
	Independent school	121	30	<5	33
	Other	182	10	<5	<5
2011–2	College	177	<5	14	<5
	Local authority	47	5	<5	<5
	Independent school	5	<5	<5	<5
	Other	39	<5	<5	<5

ALSPAC: The Avon Longitudinal Study of Parents and Children. * Includes a small number of pupils who attended Muslim faith schools; “<5” cells may include zero.

## Considerations when using the school data in ALSPAC

### Strengths and limitations of the data

One of the notable strengths of these data is the availability of faith school data linked to ALSPAC parents’ and children’s data. This unique feature presents a valuable opportunity for researchers to explore the causal relationship between characteristics of schools including type of establishment, boarding status, sex composition, and particularly childhood attendance at Christian faith schools and subsequent outcomes in physical, behavioural, and mental health, as well as academic achievement. This advantage is underscored by the dataset's capacity to minimize bias and enable the control of potential individual and parental confounders in these associations within the context of a longitudinal study. A further advantage lies in the fact that the data are not biased in regard to the source of information – for example, a parent might have been tempted to claim erroneously that the child attended a fee-paying school.

One limitation is that there are probably too few children attending single-sex schools or boarding schools for valid analysis into their advantages or disadvantages. Conversely, there are relatively large numbers of children attending Church of England and Roman Catholic faith schools for detailed analysis concerning the effects that such schooling may present. Finer detail concerning other aspects of these schools are available from questionnaires completed by the head teachers, which will be able to be used to provide further context to the basic school information described here ( Tunstall *et al.*, in preparation).

It is important to note that in this dataset, there are very few students who attended non-Christian faith schools, and these students have been merged with those who did not attend any faith schools. Among students who never attended Christian faith schools (categorized as "Non-faith" in Table 5– Table 8), only 7 had Jewish mothers, and 25 had Muslim mothers. Given that there is only one Muslim faith school and no Jewish school in Bristol, the maximum number of pupils who likely attended non-Christian faith schools is 25. Consequently, any future studies using these data will only be able to assess the association of attending Christian faith schools.

In this data note we only focused on some administrative aspects of schools such as type of establishment, boarding status, religious categories, and sex composition. However, other information about children’s education at different stages such as exam results, absence, as well as pupil characteristic data such as ethnicity, language, free school meals entitlement, and details of any Special Educational Needs (SEN) have also been linked to ALSPAC participants (e.g. Tunstall *et al.*, in preparation).

The data presented in this study is limited to England and does not encompass other regions of the UK. The dataset does not provide explanations for missing individuals, such as relocation overseas, illness, or participation in homeschooling. Additionally, consents may change over time, resulting in a decrease in the number of participants.

The value and strength of longitudinal studies, and birth cohorts in particular, lies in the data collected on multiple and repeated life course exposures. However, attrition is inevitable in large prospective, population-based studies. Traditional approaches to follow-up become increasingly challenging and expensive as the ALSPAC participants enter adulthood and potentially move out of the original area. This education data was obtained using data linkage between study participants and information held on them in the electronic records of routine national databases. Data linkage has added value to ALSPAC in several important ways. It is a cost-effective and comprehensive source of both retrospective and prospective measures of exposure and outcome status, and a tool for dealing with ‘missingness’ of data because not all participants complete all the questionnaires they are sent. It is also a form of cross-validation of data obtained from different sources and a means to obtain “objective” assessments of exposures and outcomes whose measurement is less subject to self-report or participant biases or memory losses.

## Ethical approval and consent

Ethical approval for the study was obtained from the ALSPAC Law and Ethics Committee (ALEC; IRB00003312; date: 01-06-2010) and the local research ethics committees (NHS Haydock REC: 10/H1010/70; date: 03-02-2011). The education data was provided by the DfE following their internal decision-making process, under Data Request Number DR120430.04 (dated 30-04-2012), which states that 'ALSPAC operates as a resource for the entire research community’ and 'The [education] data are pseudonymised and then used by researchers and research projects approved by the ALSPAC executive.’ Consent for the use of data collected via questionnaires (implied consent) and clinics (informed consent) was obtained from participants following the recommendations of the ALSPAC Ethics and Law Committee at the time. Questionnaires were completed in the participants’ own home and return of the questionnaires was taken as continued consent for their data to be included in the study ( Birmingham, 2018). Full details of the approvals are available from the study website. Study members have the right to withdraw their consent for elements of the study or from the study entirely at any time. When the ALSPAC children reached legal adulthood (age 18 years), the ALSPAC data linkage team, through the Project to Enhance ALSPAC through Record Linkage (PEARL), sent them 'fair processing' materials describing ALSPAC’s intention to link to their routine health and administrative data from national databases, including education, and gave them a clear means to consent or object via a written form.

## Data Availability

ALSPAC data access is through a system of managed open access. The steps below highlight how to apply for access to the data included in this data note and all other ALSPAC data. The datasets presented in this article are linked to ALSPAC project number B3397. Please quote this project number during your application. The ALSPAC variable codes highlighted in the dataset descriptions can be used to specify required variables. 1. Please read the ALSPAC access policy which describes the process of accessing the data and samples in detail, and outlines the costs associated with doing so. 2. You may also find it useful to browse our fully searchable research proposals database, which lists all research projects that have been approved since April 2011. 3. Please submit your research proposal for consideration by the ALSPAC Executive Committee. You will receive a response within 10 working days to advise you whether your proposal has been approved. If you have any questions about accessing data, please email alspac-data@bristol.ac.uk. The study website also contains details of all the data that is available through a fully searchable data dictionary. OSF: The types of school attended by the ALSPAC children from1997 to 2011 (ages 5 to 19 years): A Focus on Christian Faith Schools ( https://doi.org/10.17605/OSF.IO/ZCJ8V) ( Tohidinik *et al.*, 2024). This project contains the following extended data: do file for data note faith school.do (Stata code to derive school variables linked to ALSPAC used in this study, Stata file). CONSENT_PACK_DETAILED_BOOKLET_v3_20012011.pdf(Detailed information booklet). CONSENT_FORM_v2_11022011.pdf (Consent form). OSF: Sager checklist for article ‘The types of school attended by the ALSPAC children from1997 to 2011 (ages 5 to 19 years): A Focus on Christian Faith Schools’. https://doi.org/10.17605/OSF.IO/ZCJ8V ( Tohidinik *et al.*, 2024). Data are available under the terms of the Creative Commons Attribution 4.0 International license (CC-BY 4.0).
