# Supplementary material for: The types of school attended by the ALSPAC children from1997 to 2011 (ages 5 to 19 years): A Focus on Christian Faith Schools
Source: Wellcome Open Res. 2025 May 19;10:109. Originally published 2025 Feb 27. [Version 2] doi: 10.12688/wellcomeopenres.23025.2 (PMC12117320; doi:10.12688/wellcomeopenres.23025.2)
Supplement: Peer review history

# Reviewer report: Reviewer response for version 2

**DOI:** 10.21956/wellcomeopenres.26815.r124196

**Published:** 2025-06-25

**Authors:** Matthew H Lee

**Affiliations:** 1 Kennesaw State University, Kennesaw, USA

I believe I was asked to review this paper because of some of my publications on Christian schools (see, for example, Lee, Johnson, & Cheng, 2024 in the *Journal for the Scientific Study of Religion *and Cheng, Lee, & Djita, 2023 in the *Journal of Religion and Health*).

 The authors utilize the Avon Longitudinal Study of Parents and Children (ALSPAC), a sample of over 15,000 pregnancies that form “a longitudinal birth cohort with a wide range of genetic, psychological, physical, and environmental data” (p. 3). They link these data with various educational data sources. A tremendous amount of work has gone into recruiting the subjects, collecting the longitudinal data, and linking the data with educational data sources. The true contribution of this work will be in the future research enabled by this data collection.

 The authors could say more about the context of their work and some preliminary observations about their data. Religious schools in England receive greater public support than religious schools in other countries, notably the United States, and are also subject to greater oversight. More could be said about the context of religious schooling, financing, and accountability in England. Though, as I noted above, the primary contribution of this work will be in future research enabled by it, I believe the authors could have made a few more preliminary observations about the data, for example, the switcher rate across sectors over time, the proportion of students in each sector classified by general education or special education status, and the demographic composition of each sector.

 To reiterate, the authors make a tremendous contribution by assembling this data and making it available. It is impossible to assess the true and total contribution of their work, but one can only imagine the powerful work that can be done using this data. I look forward to seeing what insights about Christian faith schools are made possible by this data.

Are sufficient details of methods and materials provided to allow replication by others?

Yes

Is the rationale for creating the dataset(s) clearly described?

Yes

Are the datasets clearly presented in a useable and accessible format?

Yes

Are the protocols appropriate and is the work technically sound?

Yes

Reviewer Expertise:

I study the economics of education with a focus on education freedom, school choice policy, and faith-based schools.

I confirm that I have read this submission and believe that I have an appropriate level of expertise to confirm that it is of an acceptable scientific standard.

## References

[1] : How Do Parents Choose Schools for Their Children? Experimental Evidence from the Private Christian School Sector. *Journal for the Scientific Study of Religion* .2024;63(3) : 10.1111/jssr.12911 579-595 10.1111/jssr.12911

[2] : A Cross-Sectional Analysis of the Relationship Between Sabbath Practices and US, Canadian, Indonesian, and Paraguayan Teachers’ Burnout. *Journal of Religion and Health* .2023;62(2) : 10.1007/s10943-022-01647-w 1090-1113 10.1007/s10943-022-01647-w PMC943407036048354

# Reviewer report: Reviewer response for version 2

**DOI:** 10.21956/wellcomeopenres.26815.r123828

**Published:** 2025-05-28

**Authors:** Howard Worsley

**Affiliations:** 1 Trinity College Bristol, Bristol, England, UK

I am happy with the amendments made to this article

Are sufficient details of methods and materials provided to allow replication by others?

Partly

Is the rationale for creating the dataset(s) clearly described?

Yes

Are the datasets clearly presented in a useable and accessible format?

Yes

Are the protocols appropriate and is the work technically sound?

Yes

Reviewer Expertise:

NA

I confirm that I have read this submission and believe that I have an appropriate level of expertise to confirm that it is of an acceptable scientific standard.

# Reviewer report: Reviewer response for version 1

**DOI:** 10.21956/wellcomeopenres.25356.r120303

**Published:** 2025-03-25

**Authors:** Andrew O Agbaje

**Affiliations:** 1 University of Eastern Finland, Kuopio, Finland

The data note describes the characteristics of ALSPAC cohort school attendance. The data note provides tools to answer questions relating to religious exposure in childhood and adolescence and future health outcomes. Indeed other religious types are minimally represented thus questions relating to the Christian faith can be examined in detail. This is a well written and comprehensive data note, no further comment.

Are sufficient details of methods and materials provided to allow replication by others?

Yes

Is the rationale for creating the dataset(s) clearly described?

Yes

Are the datasets clearly presented in a useable and accessible format?

Yes

Are the protocols appropriate and is the work technically sound?

Yes

Reviewer Expertise:

Clinical Epidemiology, Pediatrics, Public Health

I confirm that I have read this submission and believe that I have an appropriate level of expertise to confirm that it is of an acceptable scientific standard.

# Reviewer report: Reviewer response for version 1

**DOI:** 10.21956/wellcomeopenres.25356.r120311

**Published:** 2025-03-18

**Authors:** Howard Worsley

**Affiliations:** 1 Trinity College Bristol, Bristol, England, UK

**Positives:**

 1)   I was immediately drawn to research that uses a longitudinal study to reflect on data (whether that data is already in existence or accessed for the purpose of the enquiry). Such research shows the commitment of the research team to explore a subject over time, and this shows commitment to delve more deeply into research findings and ask different questions. Such research is far more costly to researcher in terms of both time and financial investment.

 2)   This research offers a rare insight into the background culture of schools and into the controversial arena of faith schools.

**Suggestions:**

 1)   In discussing faith schools (introduction) it would be helpful to mention that there are other schools as well as Church of England and Roman Catholic schools (ie Muslim , Jewish and Sikh schools) and to detail percentages of pupils attending them. Also, the categorisation of ‘faith schools’ might alternatively be called ‘schools with a religious character’. (This is expanded subsequently in Table 3).

 2)   Say more about ALSPAC. Was this survey created for the express use of this research or are the researchers utilizing a wider research stream of data with their own scrutiny? (see Methods section).

 3)   In section ‘PEARL consent campaign’, say at what age children were ‘now considered to be young adults in their own right’.

 4)   The tables make up too much of the article and need to be further reduced. Maybe they can be part of the fuller report but for the purposes of an academic peer-reviewed article, they require further data reduction.

 5)   In the section ‘Strengths and limitations of the data’ (which I would tend to call ‘Reflection on the data’, the term ‘non-Christian faith school’ could be deemed pejorative. I would suggest saying, ‘ school which does not have a Christian religious character’.

 6)  Most importantly I feel that the article requires some tentative comment from the research team. This could be entitled, ‘Research Implications’ and offer some possible interpretation of the data. 

 For example, comment could be offered relating to the  current debate over whether ‘faith schools’ use their admissions criteria to cherry-pick more able students and to thereby show an uplift in their academic results. 

 As it stands, the article ends abruptly and is rather disappointing. It requires some conclusion whereby the research team interprets its own data.

**Typos:**

 1)  ‘Data were not extracted…’ should be ‘Data was not extracted..’ in section ‘PEARL consent campaign’.

Are sufficient details of methods and materials provided to allow replication by others?

Partly

Is the rationale for creating the dataset(s) clearly described?

Yes

Are the datasets clearly presented in a useable and accessible format?

Yes

Are the protocols appropriate and is the work technically sound?

Yes

Reviewer Expertise:

NA

I confirm that I have read this submission and believe that I have an appropriate level of expertise to confirm that it is of an acceptable scientific standard, however I have significant reservations, as outlined above.

# Response

**Published:** 2025-05-16

**Authors:** Hamid Reza Tohidinik

**Affiliations:** University of Bristol, UK

We thank the reviewer for their positive feedback and constructive comments. Our responses to each point are provided below, with the reviewer’s original comments presented in standard font and our responses in italics.

**Reviewer Comment 1:** In discussing faith schools (introduction) it would be helpful to mention that there are other schools as well as Church of England and Roman Catholic schools (ie Muslim , Jewish and Sikh schools) and to detail percentages of pupils attending them. Also, the categorisation of ‘faith schools’ might alternatively be called ‘schools with a religious character’. (This is expanded subsequently in Table 3).

***Author Response 1:*** *We updated the percentage of faith schools in England and included additional information about other types of faith schools including Muslim , Jewish, Sikh and Hindu schools.* *We agree that "schools with a religious character" is a suitable alternative. However, given its length and the frequency with which the term appears in the manuscript, we opted to use "faith schools" for the sake of brevity and readability. We referred to 'schools with a religious character' as an alternative term when first introducing faith schools in the introduction, to ensure clarity and consistency for readers.* *We also provided a brief description of faith schools and their characteristics in the introduction.* *We revised the aim of the study to clarify the specific focus on Christian faith schools.* *Reference: The Good Schools Guide. (2025) Faith schools in the UK. [Online] Available at: https://www.goodschoolsguide.co.uk/choosing-a-school/state-schools/faith-schools-in-the-uk [Accessed 4 April 2025]*

**Reviewer comment 2: ** Say more about ALSPAC. Was this survey created for the express use of this research or are the researchers utilizing a wider research stream of data with their own scrutiny? (see Methods section).

***Author Response 2:*** *The school data used in our study was not originally collected by ALSPAC for the specific purposes of our research. Instead, it was gathered through external sources and later linked to the ALSPAC data set. We have utilized this linked data to conduct our specific analysis, but it was not collected with our research question in mind. We added the following clarification to the Methods section (The ALSPAC sample and the study children): "It is important to note that the school data utilized in this analysis was not originally collected by ALSPAC. Rather, it was gathered from external sources and later linked to the ALSPAC dataset. This linkage allowed for a more comprehensive investigation and analysis of the school-related factors within the ALSPAC.".*

**Reviewer Comment 3:**  In section ‘PEARL consent campaign’, say at what age children were ‘now considered to be young adults in their own right’.

***Author Response 3:*** *Done. The updated sentence now reads: The PEARL campaign sent out a total of 13,501 consent packs to the ALSPAC young people, who were aged 18 and above (the children now being young adults in their own right).*

**Reviewer Comment 4:** The tables make up too much of the article and need to be further reduced. Maybe they can be part of the fuller report but for the purposes of an academic peer-reviewed article, they require further data reduction.

***Author Response 4:*** *We agree that in a traditional original research article, extensive tables may require reduction to enhance narrative flow. However, this manuscript is a Data Note, and as per Wellcome Open Research journal guidelines, the purpose of the article is to provide a brief description of a scientific dataset in a way that promotes transparency, accessibility, and potential reuse. Therefore, detailed tables are a central component of the article type, allowing researchers to assess the richness, structure, and utility of the data. We believe these tables are essential to meeting the aims of a Data Note and aligning with the expectations of the journal.*

**Reviewer Comment 5:** In the section ‘Strengths and limitations of the data’ (which I would tend to call ‘Reflection on the data’, the term ‘non-Christian faith school’ could be deemed pejorative. I would suggest saying, ‘ school which does not have a Christian religious character’.

***Author Response 5:*** *By "non-Christian faith school," we were referring to other types of faith schools, such as Jewish or Muslim faith schools. To improve clarity, we replaced the term with "other types of faith schools" in the text.*

**Reviewer Comment 6:** Most importantly I feel that the article requires some tentative comment from the research team. This could be entitled, ‘Research Implications’ and offer some possible interpretation of the data. 

 For example, comment could be offered relating to the  current debate over whether ‘faith schools’ use their admissions criteria to cherry-pick more able students and to thereby show an uplift in their academic results. 

 As it stands, the article ends abruptly and is rather disappointing. It requires some conclusion whereby the research team interprets its own data.

***Author Response 6:*** *We appreciate your interest in seeing a more interpretive element added to the article. While Wellcome Open Research Data Notes are intended to focus on describing the dataset and do not typically include analyses or conclusions, we recognize the value of providing context for future users. In response, we have added a paragraph offering tentative interpretation and outlining key considerations such as the role of selective admissions in faith schools and the influence of socioeconomic and demographic factors on student outcomes. We hope this addition offers helpful context to support thoughtful and informed use of the dataset in future research.*

**Reviewer Comment 7:** Typos:

 ‘Data were not extracted…’ should be ‘Data was not extracted..’ in section ‘PEARL consent campaign’.

***Author Response 7:*** *Thank you for pointing that out. We have reviewed the sentence and kept "Data were not extracted..." as it aligns with the standard use of "data" as a plural noun in scientific writing. However, we’re happy to revise it if the journal prefers "data" to be treated as a singular noun.*
